# Segmentation Based on Attitudes Toward Corporate Social Responsibility in Relation to Demographical Variables and Personal Values – Quantitative and Qualitative Study of Polish Consumers

**DOI:** 10.3389/fpsyg.2020.00450

**Published:** 2020-03-17

**Authors:** Aleksandra Furman, Dominika Maison, Katarzyna Sekścińska

**Affiliations:** Department of Business Psychology and Social Innovations, Faculty of Psychology, University of Warsaw, Warsaw, Poland

**Keywords:** corporate social responsibility, segmentation study, personal values, demographical variables, qualitative study

## Abstract

The purpose of the present research was to create market segmentation of Polish consumers that would capture differences in reactions to Corporate Social Responsibility (CSR), taking into account sociodemographic data and consumers’ value structure. In order to better understand the extracted segments, a mixed method approach was adopted. The first quantitative study was conducted on a nationwide representative sample of Poles aged 18–55 years (*N* = 1055, CAWI survey). A subsequent qualitative stage covered 24 semi-structured in-depth individual interviews, with representatives of each segment identified in Study 1. Consequently, six segments of Poles were extracted and described, differing in knowledge, attitudes and beliefs about CSR: Sensible Optimists (15%), Sensitive Intellectuals (18%), Family Pragmatics (21%), Passive Poseurs (19%), Excluded and Frustrated (12%) and Corpo-Egoists (15%). The study showed both demographic and psychological differences in between segments. Segments with positive attitudes toward CSR are more female. Segment of least positive attitudes is manly and youngest one. However, results for age, education level and economic status are less conclusive. Personal values proved to be more useful in understanding different attitudes toward CSR than demography. Segments that are more open to CSR prize self-transcendence and maturity values, while less open segments are more oriented toward social status values.

## Introduction

In recent years, a meaningful change can be observed in business’ approach to its goals and modes of functioning, as the concept of Corporate Social Responsibility (CSR) has increased in popularity. Many international corporations, in cooperation with external partners, undertake activities that use the business’s potential in order to help solve social and environmental problems. Consequently, business goes beyond the basic scope of its activity aimed at multiplying profits. However, many companies have discovered that a strategic approach to social responsibility can also be an important element of building market competitiveness (Porter and Kramer, 2006; Kim et al., 2010; Inoue and Kent, 2014).

However, an effective CSR strategy requires positive consumer response. The growing body of literature focuses on consumers’ reactions to CSR, describing their market and individual basis. Many studies concentrate on variables that modify consumers’ reactions, yet are connected with companies, such as reputation; Yoon et al., 2006; Elving, 2013), corporate fit (Becker-Olsen et al., 2006; Nan and Heo, 2007; Elving, 2013), stated firm’s motives (Forehand and Grier, 2003; Becker-Olsen et al., 2006; Chernev and Blair, 2015). Less interest is shown toward more internal, psychological variables. There is evidence that consumers react to CSR differently depending on their demographic characteristics (Meyers-Levy, 1989), knowledge and the personal importance of CSR (Bhattacharya and Sen, 2004), as well as personal values (González-Rodríguez et al., 2016). However, few studies have been conducted in post-communist countries (González-Rodríguez et al., 2015), although scholars admit that there might be a vast discrepancy in CSR politics and social reactions to it between Eastern and Western European countries due to differences in political and economic history (Matten and Moon, 2008).

The goal of the present study is to give a complex and in-depth view of Polish consumers’ approach to CSR. Present market segmentation, in contrast to prior qualitative typologies (Mohr et al., 2001) and quantitative segmentation studies in this area (Vassilikopoulou et al., 2005) is more comprehensive: in our study we include not only a rich set of variables connected to knowledge and opinions of CSR and demographic variables, but also personal value structure, which enables us to understand why certain segments are apt to engage in CSR more than others. Additionally, in order to give a more complex description of different attitudes to CSR as well as a possible explanation of mechanisms underlying such reactions, a mixed method approach is used (Teddlie and Tashakkori, 2003). In effect, we succeed not only in explaining what makes consumers’ approach to CSR more or less positive, but also in capturing various unique patterns of reactions. We are also able to describe the psychological basis of different attitudes toward CSR.

## Literature Review

### The Concept of CSR

There are many definitions of CSR and many approaches to systemize it (Wang and Juslin, 2011; Sarkar and Searcy, 2016). For the purposes of our study we will follow the definitions by Carroll (1991) and more recent, by European Commission (2011). Carroll (1991) states that “The total corporate social responsibility of business entails the simultaneous fulfillment of the firm’s economic, legal, ethical, and philanthropic responsibilities” (p. 43). European Commission (2011) characterizes CSR as “the responsibility of enterprises for their impact on society” which can be fully realized by following the law and integrating “social, environmental, ethical, human rights and consumer concerns into their business operations and core strategy” (p. 6). Both definitions embrace firms’ voluntary and legally mandated actions, in all possible areas, such as production, workplace, environment, and society. Both definitions also imply that CSR is a result of a company’s broader strategy, the way it sees its own responsibility to the environmental and social background.

### Factors Underlying Consumers’ Reactions to CSR

#### Knowledge, Attitude Toward CSR and Responsible Consumption

One important individual variable influencing individual reactions to firms’ social engagement is what consumers know and their opinions of an idea of corporate social responsibility. Scholars indicate that in order to evoke positive consumer reactions, consumers must have some understanding of an idea of CSR and CSR-related companies’ actions (Pomering and Dolnicar, 2009). Generally, the greater the awareness of CSR is, the better the reactions are (Bhattacharya and Sen, 2004; Kim, 2019). Additionally, a positive personal attitude toward CSR enhances consumers’ positive reactions to such engagement, such as improvement of brand awareness, better attitude and attachment to a company (Sen and Bhattacharya, 2001; Bhattacharya and Sen, 2004), purchase intention (Creyer, 1997) or diminishing negative reactions to a firm’s crisis (Klein and Dawar, 2004).

It is often assumed that consumer social responsibility serves as a base for corporate social responsibility – specifically, the idea of corporate engagement is more important to consumers who are more socially responsible themselves (Devinney et al., 2006; Caruana, 2007; Caruana and Chatzidakis, 2014). Consumer social responsibility is expressed through taking into account whether a firm acts responsibly when shopping (Öberseder et al., 2011) and enhanced purchase intention of products of responsible companies (Creyer, 1997; Bhattacharya and Sen, 2004). However, this is just one aspect of responsible consumption. Others are shopping in such a way that minimizes the impact on the environment or engaging in prosocial actions organized by charities or other institutions (Small and Cryder, 2016).

Another important issue is the perceived motives for a company’s engagement – whether it is driven externally (for example, in pursuit of bettering a firm’s reputation), or internally (out of genuine concern for the problem). Perceiving motives as purely external diminishes positive reactions to the firm’s engagement (Chernev and Blair, 2015; Habel et al., 2016) or might even backfire when a firm has bad reputation (Yoon et al., 2006). Most research assumes that motives are an objective characteristic of a company’s engagement, resulting from specific actions or how they are communicated (Becker-Olsen et al., 2006; Yoon et al., 2006). Thus, perceived motives are often a manipulated variable in experimental studies. However, a study done by Forehand and Grier (2003) shows that when not primed, consumers do not always consider the firm’s motives. What is more, it is possible that motive perception not only results from what is said or shown by the company, but is also a consequence of individual traits and attitudes toward CSR and more generally toward all societal surroundings. An individual who is less prosocial may project his own attitudes on the company and perceive it quite differently than more other-oriented persons. Additionally, there is also an issue of maturity of both the company’s actions and consumer perception. According to the cited definition of CSR by Carroll (1991), the CSR should be strategic and should concern all of the company’s responsibilities. Such politics should go beyond one-time actions aiming at quick reputation repair. Thus, perceiving a firm’s motives as less complex and purely extrinsic might be a result of immature CSR politics, but may also be connected with a less advanced, simpler understanding of the idea by consumer.

Based on the presented assumptions, we include both knowledge of and attitude toward CSR, personal responsible consumption level as well as perceived motives of companies’ engagement as individual characteristics, serving as a base for segmentation analysis.

#### Demographical Variables

Numerous studies have revealed the significant role of demographical variables in explaining consumers’ reactions to CSR. Usually, women are more sensitive to CSR communication and more interested in products and companies engaged in good causes (Meyers-Levy, 1989; Skoe et al., 2002; Moosmayer and Fuljahn, 2010; Scharma et al., 2012; Wang and Juslin, 2012; González-Rodríguez et al., 2014). Age also shows significant, though less consistent effects. Some evidence shows that young people are more open to corporate responsibility than older consumers and they increasingly expect it from business (Dickson, 2001; Diamantopoulos et al., 2003; Elias, 2004). However, there are also contradictory findings, such as the study of Vassilikopoulou et al. (2005), indicating that a positive attitude to CSR increases with age. Additionally, education level (Kelley et al., 1990; Roberts, 1996; Diamantopoulos et al., 2003) and economic status (Roberts, 1996) are reported to have a positive correlation with attitudes toward CSR.

However, including demographic variables does not always give significant results. For example, the results of Diehl et al. (2013), exploring reactions to CSR advertisement, revealed negative relationship between reaction to CSR appeal and age, however, it was only significant in one of four tested countries. Furthermore, there was no relationship found between reaction to CSR advertisement and education level. Other authors suggest that studies exploring the relationships between demographical variables such as age or income and perception of CSR or pro-ecological attitudes often give insignificant or opposite results due to the fact that the actual relationship is curvilinear (Tian et al., 2011; Park et al., 2012).

There are also numerous studies showing that it segmentation analyses, it is advisable to include also other than demographic variables. Pérez et al. (2015a) find no relations between gender, age, or education level and perception of CSR, which leads them to conclude that demographical variables are useless in segmenting consumers in this context. Also Straughan and Roberts (1999) in their green behavior segmentation of students found demographic criteria as less useful in profiling, while psychological variables, such as perceived self-effectiveness or altruism as more effective in explaining the variation. In fact, the usefulness of demographic variables in market segmentation analyses has been broadly discussed across other fields. Segmentation studies based on not only demographic, but also psychographic variables has been reported as more useful in a variability of fields, such as planning market positioning strategies (Lin, 2002), creating social campaigns (Boslaugh et al., 2005), and tourist places marketing (Andereck and Caldwell, 1994). Tkaczyński et al. (2009), based on the broad analysis of different approaches toward tourist market segmentation, recommend the combined approach – using not only one kind of variables, such as demographic, but also including behavioral or psychographic ones, as most useful.

In the light of the results cited above it seems clear that, in spite of relations between demographic variables and CSR perception, in order to deeply understand and predict consumers’ reactions to corporate responsibility, it is necessary to investigate the role of more psychographic variables, such as values, needs, motivation and personality structure (Fennell, 1997; Maison, 2019).

#### Personal Values

Personal values, understood as ‘important transsituational goals, (…) that serve as guiding principles in the life of a person’ (Schwartz, 1994, p. 21), are an important factor when discussing personal attitudes to firms’ prosocial engagement and personal relevance of corporate responsibility.

Numerous studies have indicated that people’s values have a significant impact on their ethical consumption behavior (Dickson, 2000, 2001; Nonis and Swift, 2001; Chernev and Blair, 2015). Anderson and Cunningham (1972) found that dogmatism, conservatism, status consciousness, cosmopolitanism, personal competence and alienation were related to ethical consumer behavior. Results of a segmentation study of consumer attitudes to fair-trade coffee (Pelsmacker et al., 2005) showed that a segment most open to fair-trade (fair-trade lovers) was high on conventional values. Studies based on Shwartz’s values theory (1994) indicate that people who prize self-transcendent values react to CSR campaigns more positively (González-Rodríguez et al., 2014, 2015; Habel et al., 2016). Usually, conservation values also correlate positively with CSR attitudes and ethical consumption (González-Rodríguez et al., 2014, 2015; Habel et al., 2016). However, Wang and Juslin (2009, 2011) studies on Chinese students’ personal values only reveal a partially consistent pattern. A group of students with high self-transcendent values were more sensitive to CSR, however, students with high conservation values were less sensitive. Another important dimension is materialistic and power values, usually related to less ethical consumer behaviors (Wang and Juslin, 2011; González-Rodríguez et al., 2015). A study conducted by Unanue et al. (2016) shows that people focused on intrinsic life goals, such as strengthening relationships (associated with values like universalism and benevolence) and personal development (related to openness to experience, and self-direction) more often undertook various pro-environmental activities, such as driving less or boycotting companies negatively affecting the environment. Moreover, people with extrinsic life goals (associated with self-enhancement values) were far less likely to engage in pro-environmental initiatives.

All the above evidence provides theoretical premises to include personal values in segmentation studies concerning attitudes to CSR. Overlooking the psychographic characteristic of the segments, such as personal values is a limitation of to-date studies. Previous segmentation research in this area concentrated mostly on CSR-related and demographic variables (Vassilikopoulou et al., 2005; McFadden et al., 2013; Pérez et al., 2015b). Including personal values will make it possible to not only capture a variety of attitudes and reactions to CSR, but also to understand their psychological premises.

## Materials and Methods

The main goal of the present research was market segmentation of Polish consumers, based on their CSR knowledge and attitudes, demographic variables and personal values. In order to better understand the psychological specifics of each segment, secondary qualitative study was carried out.

### Study 1 – Quantitative Segmentation of Polish Consumers

#### Method and Participants

A nation-wide representative survey was conducted. A random-quota sample following the demographic structure of the Polish population aged 18–55, in respect to gender, age and size of residence was constructed from an online panel (CAWI – Computer Assisted Web Interview). A total number of 1055 participants completed the survey: 514 women, 541 men, aged 18-55 (*M* = 33.39, *SD* = 10.52). The detailed demographic characteristic of the sample is presented in Table 1. At the beginning of the computer task participants were asked to participate in the study and were told that they could stop at any moment. They were also informed that the study was anonymous and the data would be analyzed at a group level. The study was carried out in accordance with the recommendations of the ethics committee of the Faculty of Psychology, University of Warsaw.

**TABLE 1:** Demographical characteristics of the quantitative study sample.

**Variable**	***N***	**%**
**Sex**		
Female	514	48.7
Male	541	51.3
**Age**		
18–24 y.o.	265	25.1
25–34 y.o.	325	30.8
35–44 y.o.	248	23.5
45–55 y.o.	217	20.6
**Size of the place of living**		
Villages	359	34.0
Small towns (up to 20k citizens)	134	12.7
Medium towns (20–99k citizens)	203	19.2
Cities (100–500k citizens)	219	20.8
Metropolies (more than 500k citizens)	140	13.3
**Education**		
Primary	34	3.2
Vocational	105	10.0
High school	558	52.9
Higher	358	33.9
**Marital status**		
Single	444	42.1
Living with partner	120	11.4
Married	422	40.0
Divorced	58	5.5
Widowed	11	1.0

#### Measurements

The first part of the survey investigated opinions, attitudes and behaviors toward CSR. Consumers’ perceived level of knowledge of CSR was measured using a scale concerning four concepts: corporate social responsibility, cause-related marketing, fair trade and sustainable development. Participants chose one of three answers: (a) I have heard of it and I know what it means, (b) I have heard of it, but I don’t know what it means, (c) I haven’t heard about the concept.

Personal attitude toward CSR was measured with two questions. The first captured the extent to which responsibility is expected of companies with a choice of one of four perceived objectives of the existence of companies: (1) The main goal of companies is to make money – I do not expect any other action from them; (2) Companies are primarily there to make money, but should also take some action on the environment, communities, etc.; (3) Companies should actively support various activities for the benefit of society and the environment, e.g., allocating their money and time for those purposes; (4) Companies should not only support, but also initiate various activities for the benefit of society and the environment, and actively seek solutions to solve various problems. The second question concerned consumers’ emotional attitude toward CSR: “What is your reaction when you hear that a company is engaged in societal or environmental issues?” (answer on a 3-point scale: I have positive feelings, I don’t care, I have negative feelings).

Responsible consumption was measured by frequency of involvement in 19 different activities. The respondents assessed the frequency of performing any of the listed activities in the last year on a 4-point scale (0- never, 1- once, 2- a few times, 3- many times). Exploratory factor analysis revealed a three-factor structure: pro-CSR activities (e.g., buying products mainly for ethical reasons), pro-ecological activities (e.g., segregation of waste) and prosocial engagement (e.g., donations on the street).

Perceived motives of engagement were assessed using a 12-item, 4-point scale. Respondents assessed the extent to which each of 12 possible motives for firms’ engagement was in his/her opinion valid. Exploratory factor analysis revealed a three-factor structure, comprising motives lining up from most extrinsic to most intrinsic: pure profit (e.g., They want to reduce business expenses, They do it mainly for money, to earn more), immediate image benefits (e.g., Firms do it in order to enhance reputation and public image, They want to gain more trust), and long-term, win-win strategy (e.g., They just want to engage, solve the problem, It increases the loyalty of the employees, It helps to build market competitiveness).

The second part of the survey measured personal values and demographic variables. Personal value structure was measured using a 38 item, 5 point Likert-type scale. Values were chosen from the Rokeach and Shwartz values inventories, based on their relatedness to CSR (Rokeach, 1973; Schwartz, 1994). Exploratory factor analysis revealed a 5-factor structure, comprising: self-transcendence and maturity (e.g., care for others, love, true friendship, justice, tolerance, honesty, reliability, freedom, wisdom); social image (e.g., humility, care for good opinion, being helpful), security (e.g., safety of the nation, world peace, social order), openness (e.g., creativity, curiosity) and social status (e.g., social dominance, influence, wealth).

All the scales described above are available in Supplementary Appendix.

At the end of the study the participants answered questions about their sex, age, education level, socio-economic status, including net income, marital status and having children.

### Analysis

The data obtained were first subjected to a factor analysis in order to limit the number of variables introduced in factor analysis. As a second step, a cluster analysis was conducted, using k-means cluster analysis method (Haughton et al., 2009). Afterward, the segments were characterized and named based on careful analysis of features that differentiate them from the rest of the population (Weinstein, 1994).

### Study 2 – Qualitative Investigation of Psychological Differences Between Segments

#### Method and Participants

Study 2 comprised 24 semi-structured in-depth individual interviews, with representatives of each segment extracted in Study 1. Fourteen women and ten men participated in the study. Interviews were conducted in two locations, differing in size: Warsaw (over 1.750 million citizens) and Krosno (close to 50 thousand citizens). The characteristics of each respondent are presented in Table 2. Participants received a remuneration of PLN 80 (USD 20) for participation in the study. Each interview lasted 2 h and was recorded.

**TABLE 2:** Demographical characteristics of the qualitative study sample.

**Segment**	**Sex**	**Age**	**Profession**	**Place of living**
Sensible Optimists	Female	40	Babysitter	Warsaw
	Male	43	Coach	Warsaw
	Female	46	Teacher	Krosno
	Female	52	Accountant	Warsaw
Sensitive Intellectuals	Female	27	Physiotherapist	Warsaw
	Female	32	Quality control employee	Krosno
	Female	32	Shop assistant	Krosno
	Female	33	Secretary	Warsaw
	Female	35	Kindergarten teacher	Warsaw
Family Pragmatics	Female	33	Unemployed	Krosno
	Male	41	Solicitor	Warsaw
	Female	66	Pensionary, earlier factory worker	Krosno
Passive Poseurs	Male	38	Consultant, business owner	Warsaw
	Female	40	Clinic owner	Warsaw
	Male	40	Musician	Warsaw
	Female	44	Beautician	Warsaw
	Male	51	Unemployed	Krosno
Excluded and Frustrated	Female	24	Student	Warsaw
	Male	46	Unemployed	Krosno
	Female	50	Carer for the elderly	Krosno
Corpo-Egoists	Male	20	Student	Krosno
	Male	23	Student, psychotherapy intern	Warsaw
	Male	25	Student, hospice worker	Warsaw
	Male	30	b-to-b corporation dealer	Warsaw

#### Materials

The interview guide concerned life priorities and values, undertaking charitable, pro-ecological and civic activities, knowledge and attitudes toward Corporate Social Responsibility, perceived motives for the companies’ engagement, the need for business involvement in various areas and interest in CSR communication, as well as the extent to which they consider the positive and negative impact of companies on their surroundings when choosing products and services.

#### Analysis

The qualitative results were interpreted in accordance with the thematic analysis guidelines (Braun and Clarke, 2006). Transcripts from interviews were submitted to a coding procedure in search of common, repeating threads. Next, the identified codes were grouped into superior themes, repeated both in individual interviews and between interviews. The themes thus obtained have been defined and described.

## Results

### Quantitative Differences Between the Segments

Based on cluster analysis, six segments, which were very different between and coherent within, were extracted. In order to check differences on key dimensions included in the model, a set of ANOVA and χ^2^ tests were conducted.

#### Knowledge of CSR

The χ^2^ showed significant differences in declared knowledge of the concepts of CSR; χ^2^(10) = 101.28; *p* < 0.001; *Cramer’s V* = 0.22, fair-trade; χ^2^(10) = 116.62; *p* < 0.001; *Cramer’s V* = 0.24, cause-related marketing; χ^2^(10) = 99.58; *p* < 0.001; *Cramer’s V* = 0.22 and sustainable development; χ^2^(10) = 121.23; *p* < 0.001; *Cramer’s V* = 0.24. Segment 1 declared greater knowledge than expected of all tested concepts. Segment 2 declared better than expected knowledge of the concepts of fair-trade and sustainable development. Segment 3 and 5 knew all tested concepts less often than expected (| standardized residual| > 1.96).

#### Attitude Toward CSR

The χ^2^ showed significant differences in expectations of firms; χ^2^(15) = 230.25; *p* < 0.001; *Cramer’s V* = 0.27. Segment 1, 2, and 3 expressed the notion that firms should only make money and that they do not expect anything else of them less often than expected, while Segment 4 and 6 chose such statement more often than expected (| standardized residual| > 1.96).

Also, emotional attitudes toward CSR were significantly different; χ^2^(10) = 300.03; *p* < 0.001; *Cramer’s V* = 0.38. Segments 1 and 2 declared reacting with positive emotions more often than expected, while positive reactions occurred less often in Segments 4, 5, and 6 (| standardized residual| > 1.96).

#### Responsible Consumption

An ANOVA test revealed differences between extracted segments in terms of pro-CSR activities (*F*[5,1049] = 35.06, *p* < 0.001), pro-ecological activities (*F*[5,1049] = 18.43, *p* < 0.001) and prosocial engagement (*F*[5,1049] = 35.38, *p* < 0.001). Means and SDs for each group are presented in Table 3.

**TABLE 3:** CSR-related, psychological and demographical characteristics of the six segments.

	**Segment 1**	**Segment 2**	**Segment 3**	**Segment 4**	**Segment 5**	**Segment 6**
Knowledge of CSR: declaring knowledge of what CSR means (%)	35.7	24.3	4.1	24.2	11.8	22.3
Expectations of firms: I believe that firms should only make money and I do not expect any engagement (%)	3.2	3.7	9.6	34.8	22.8	45.2
Positive emotional attitude toward CSR: experiencing positive emotions when hearing of a company’s CSR (%)	91.7	90.5	72.0	42.0	45.7	22.3
Responsible consumption: pro-CSR activities *M(SD)*	4.1 (0.4)	3.9 (0.5)	3.8 (0.5)	3.7 (0.6)	3.6 (0.6)	3.4 (0.5)
Responsible consumption: pro-ecological activities *M(SD)*	4.2 (0.4)	4.1 (0.5)	4.0 (0.5)	3.8 (0.7)	3.8 (0.6)	3.7 (0.5)
Responsible consumption: prosocial activities *M(SD)*	4.2 (0.5)	3.9 (0.6)	3.9 (0.5)	3.7 (0.7)	3.7 (0.7)	3.2 (0.7)
Perceived motives for engagement: pure profit *M(SD)*	3.1 (0.5)	3.1 (0.4);	3.0 (0.4);	2.9 (0.5)	2.9 (0.6)	3.1 (0.6)
Perceived motives for engagement: immediate image benefits *M(SD)*	3.2 (0.4)	3.2 (0.4)	3.1 (0.3)	2.9 (0.5)	2.9 (0.6)	3.1 (0.5)
Perceived motives for engagement: long-term, win-win strategy *M(SD)*	2.9 (0.4)	2.8 (0.4)	2.8 (0.4)	2.8 (0.5)	2.5 (0.5)	2.3 (0.5)
Values: self-transcendence and maturity *M(SD)*	4.67 (0.3)	4.41 (0.4)	4.44 (0.4)	4.00 (0.6)	4.12 (0.7)	4.28 (0.4)
Values: social status *M(SD)*	3.82 (0.5)	3.69 (0.6)	3.67 (0.5)	3.79 (0.6)	3.62 (0.6)	3.72 (0.6)
Values: social image *M(SD)*	4.05 (0.6)	3.77 (0.5)	3.94 (0.5)	3.78 (0.6)	3.53 (0.7)	3.42 (0.7)
Values: openness *M(SD)*	4.32 (0.4)	4.06 (0.5)	3.94 (0.4)	3.88 (0.6)	3.76 (0.6)	4.01 (0.5)
Values: security *M(SD)*	4.38 (0.5)	4.04 (0.5)	4.15 (0.5)	3.77 (0.6)	3.77 (0.7)	3.57 (0.7)
Sex: men (%)	39	35	42	66	57	73
Age *M(SD)*	37.1 (10.7)	29.6 (7.9)	43.8 (7.3)	29.0 (8.9)	29.3 (8.7)	28.9 (8.7)
Education:% with higher education	47.5	74.6	5.5	20.7	20.5	38.9
Monthly net income per capita in PLN (USD)	1674 (399)	1334 (318)	949 (226)	1041 (248)	833 (198)	1686 (400)

A set of *post hoc* Tamhane tests revealed that Segment 1 showed significantly more, and Segment 6 significantly fewer pro-CSR activities than all other segments. Segment 5 showed significantly fewer pro-CSR activities compared to all segments apart from Segment 4. There were no significant differences between Segments 2, 3, and 4. In terms of pro-ecological activities, Segment 1 showed significantly more such activities than all segments apart from Segment 2. Segment 2 presented more pro-ecological engagement compared to other groups apart from Segments 1 and 3. Segment 3 showed less engagement than Segment 1, but more than Segments 4, 5, and 6. Segments 4, 5, and 6 did not differ from each other. In terms of prosocial engagement, Segment 1 showed significantly more, and Segment 6 significantly fewer prosocial activities than all other segments. Segment 2 showed fewer prosocial activities than Segment 1, but more than Segments 5 and 6. Segment 3 presented less prosocial engagement than Segment 1, but more than Segments 4, 5, and 6. Segments 4 and 5 did not differ from each other (*p*-value level < 0.05).

#### Perceived Motives of Engagement

An ANOVA test revealed differences between extracted segments in terms of perceived motives of engagement: pure profit (*F*[5,1049] = 6.16, *p* < 0.001), immediate image benefits (*F*[5,1049] = 18.95, *p* < 0.001) and long-term, win-win strategy (*F*[5,1049] = 38.70, *p* < 0.001). Means and SDs for each group are presented in Table 3.

*Post hoc* Tamhane tests showed that Segment 4 perceived firms as profit-driven significantly less often than other segments. Segment 1 and 2 attributed reputation-driven motives more often than all other segments apart from Segment 6. Segment 3 viewed firms as reputation-driven less often than Segments 1 and 2, but more often than Segment 4. Segment 6 attributed reputation motives more often than Segments 4 and 5. Segment 1 viewed CSR as strategy-driven more often than all other segments apart from Segment 4. Segment 6 viewed CSR as a long-term, win-win strategy significantly less often than all other groups, and Segment 5 less often than other groups apart from Segment 6. Segment 2, 3, and 4 did not differ (*p*-value level < 0.05).

#### Values

An ANOVA test revealed differences between extracted segments in terms of values: self transcendence and maturity (*F*[5,1049] = 56.54, *p* < 0.001), social image (*F*[5,1049] = 23.39, *p* < 0.001), security (*F*[5,1049] = 22.88, *p* < 0.001), openness (*F*[5,1049] = 16.07, *p* < 0.001) and social status (*F*[5,1049] = 6.78, *p* < 0.001).

*Post hoc* Tamhane tests showed that Segment 1 valued self-transcendence and maturity values significantly more than all other segments. Segment 2 esteemed these values more than Segments 4, 5 and 6. Segments 4 and 5 valued self-transcendence and maturity values significantly less than the rest of the segments. Social image values were highly esteemed by Segment 1 (significantly more than all other segments) and Segment 3 (significantly more than Segment 2, 5, and 6) and less valued by Segment 6 (significantly less than all other segments) and Segment 5 (significantly less than all other segments apart from Segment 6). Segment 1 and 2 valued openness more than other segments. This group of values was least esteemed by Segment 5 (significantly less than Segment 1, 2, and 6). Security values were most esteemed by Segments 1 and 3 (significantly more than by other segments). Segments 4, 5, and 6 valued security significantly less than Segments 1, 2, and 3, with no significant differences in between them. As far as social status values are concerned, there was only one significant difference found: Segment 1 esteemed these values higher than Segment 5.

### Characteristic of the Segments (Based on Quantitative and Qualitative Study)

Descriptions below embrace demographical, psychological and CSR-related differences between the segments based on quantitative data (Table 3). Each description ends with qualitative insights and quotations illustrating the specifics of each group.

#### Segment 1 – Sensible Optimists (15%)

Well-educated working professionals. Slightly more women than men, married, with children. Sensible Optimists value self-transcendence and openness more than other segments. Most are aware of CSR, know what it is, expect these kinds of activities from companies, and perceive firms’ engagement as intrinsically motivated. They show the highest self-engagement in all areas: ecological, social, and CSR. The qualitative interviews with this group revealed their attentive and rational concern for social problems. They are optimistic, believing that the world might be changed by individual choices and feel morally obliged to make the right choices themselves. Their engagement is rational and strategic – they consider whether their actions might make real differences. They feel that companies also should look for areas they might improve in their surroundings and also should be strategic in their actions.

“When I make decisions on support, I try not to do it impulsively. This is a terrible situation, when the child is ill, but if you wanted to reach into your pocket every time you feel compassion, you would soon become a person in need yourself. I help in random situations, such as a flood, a hurricane, and here I am looking for systemic solutions, (.) I vote for projects from civic budgets, I take part in elections.” Fulfilled Optimist, female, 40 y.o.

*“If a company* (…*) does not respect employee rights, even if it transfers hundreds of millions for social purposes, there is still no basis to consider such action in terms of responsibility at all.” Fulfilled Optimist, male, 43 y.o.*

#### Segment 2 – Sensitive Intellectuals (18%)

Mostly female segment (65%), best-educated. Many not married yet, without children. Similarly to the previous group, they value self-transcendence and maturity most. Compared to Sensible Optimists, they declare less knowledge of CSR (however, still greater relative to the following segments), but when they hear about firms’ engagement, they react with positive emotions, they perceive motives for such engagement as complex and internal and are likely to engage. They believe business should act in the interest of society and the environment. Only the qualitative results showed differences compared to the preceding segment and the uniqueness of Sensitive Intellectuals. It appears that their willingness to help is based rather on their sensitivity and empathy than on rational thoughts and strategic plans. They are willing to engage whenever they are moved, without taking into consideration whether such actions would really have an impact on solving the problem. This line of thinking is projected on assessments of companies’ engagement. First, Sensitive Intellectuals expect – and perceive – engagement to be internally driven. Second, for Sensitive Intellectuals, the personal relevance and emotional magnitude of the problem, and not the social impact, is what matters most.

“*Sometimes when I see someone on the street collecting money, I give him some, out of feeling sorry for him. Even if I know that sometimes they might be pretending*.” *Sensitive Intellectual, female, 27 y.o.*

“If a company earns and has money, but it does not get involved in anything, I’m bothered. Behind the company there are always people and I do not want to buy from, support those who have no empathy themselves, do not see what is going on around them and are only focused on their own profit.” Sensitive Intellectual, female, 33 y.o.

#### Segment 3 – Family Pragmatics (21%)

The oldest, least-educated segment. They value self-transcendence values, however, no more than social image and security values. It is important for them to take good care of their close ones, feel safe, acquire a stable position in life as well as to be liked and trusted by others. They admit their knowledge of CSR is poor, and they do not expect such engagement by the companies. However, they declare mostly positive emotional reactions when hearing of CSR engagement of the company. They engage mostly in pro-ecological activities, but it is more because of the direct benefits to themselves (reducing costs) than out of concern for the environment.

The qualitative study revealed that Family Pragmatics are likely to engage in all sorts of initiatives that have direct, visible effects on their close environment. The tangibility of the results of the action has key importance to them. When an action is vague to them, they do not have enough concrete information or do not see the results, they are easily skeptical about the effectiveness of an action and the company’s motives and choose not to engage.

*“I’m getting involved at my daughters’ school. I go on school trips, I help to organize performances. I also get involved in the local community, for example, the Neighbor’s Day. Everyone brought something* (…*), and this event has made us get to know each other.” Family Pragmatic, female, 33 y.o.*

“I do not think there are companies that just want to do good. It is common knowledge that nobody does anything for free. Nobody will be so good.” Family Pragmatic, female, 33 y.o.

#### Segment 4 – Passive Poseurs (19%)

Most often young men, blue-collar workers. Compared to other segments, they show quite level pattern of value preference, which might be due to self-presentation efforts – they strive to be perceived as more caring and prosocial than they really are. They declare knowledge of CSR comparable to Sensitive Intellectuals, however, react significantly less often with positive emotions and more often with negative ones to a firm’s engagement. One third believes that firms should exclusively make money. Their pro-ecological and prosocial engagement compared to Segments 1, 2, and 3 is low.

The qualitative stage revealed the vast discrepancy between Passive Poseurs’ declarations and actions. Most of them underline the importance of being responsible and helpful to others, they often build complicated narratives of when and how they should engage prosocially, however, when it comes to talking about actual actions, they vainly strive not to show a lack of any real engagement. When it comes to CSR, they often declare openness to such firms’ actions – however, this declaration would not be followed by any personal engagement, such as buying the product or even thinking better of a company – Passive Poseurs are easily skeptical and assign self-oriented motives to a firm.

“I have a strong need and awareness that engaging in prosocial activities has value on many levels: personal, emotional level, creating structures based on people, not on politics, social processes, improving our lives, etc. [.] On the other hand, I regret how few things I can do as a citizen.” Passive Poseur, male, 40 y.o.

“I am resistant to the CRM messages on the product. It does not work on me.” Passive Posseur, male, 38 y.o.

#### Segment 5 – Excluded and Frustrated (12%)

Young segment, mostly men, many of them from villages, poorly educated and often unemployed, in the worst financial situation and least satisfied with life. Their knowledge of CSR is poor and they usually declare that a firm’s engagement does not trigger any emotional reaction. They perceive companies’ motives for engagement as simple, connected with direct benefits. However, they declare not expecting any engagement from a company less often than other CSR-negative segments (Segment 4 and 6).

The qualitative study revealed Excluded and Frustrated’s lack of knowledge not only of CSR, but also of many other less innovative mechanisms of social and ecological problem solving, such as rules for waste segregation. Moreover, they are not interested in gaining more knowledge. They see the world as divided between the weak and the strong. They perceive themselves as the weak and do not see why they should help others while they could use help themselves. They strive to acquire a sense of stability in their lives and envy those who have more luck and have better social and material positions. On the other hand, they perceive companies as the strong and believe they should share their resources with others. However, they do not believe that firms really want to do that and doubt their intentions. They do not understand their role as the consumers in such companies’ actions and certainly would not consider them as a factor in purchasing products.

“*Helping others? No! I started a family at the age of 23*, (…*) it had to be taken care of, the first child, the second child. I did not have time to do something bigger.* (…*) Generally, such actions are good, but I did not have time.” Excluded and Frustrated, male, 46 y.o.*

“Companies are there to make money, and not for anything else. But they should get involved, they have money, they may help.” Excluded and Frustrated, female, 50 y.o.

#### Segment 6 – Corpo-Egoists (15%)

Mostly male (73%), young, from the biggest cities, mainly students or white-collar workers with high incomes. They are most often single. Like Sensible Optimists, they value self-transcendence, maturity and openness, but unlike Segment 1, they do not value social image. They declare being knowledgeable about CSR, but have the least positive attitudes. Almost half does not expect any kind of engagement of companies and the vast majority (73% compared to 36% for all segments) declares no emotional reaction when hearing of a firm’s engagement. They also present the greatest level of skepticism toward companies’ engagement and do not believe their motivation is altruistic. They show the least personal involvement in prosocial, pro-ecological and pro-CSR actions.

The qualitative study showed that values of striking importance to Corpo-Egoists are freedom, self-development and self-reliance. As they do not give much concern to others on their way to self-development, they do not expect that from companies either. Moreover, they often see too much concern for social and environmental problems as ethically ambiguous, because it serves as an obstacle to development, which in turn might bring huge benefits for humanity. They are egocentric and not willing to help others. They support major system changes, not small changes one could make to his or her close surroundings.

“I’m not for collecting money in the street, this is not how it should be. I believe that a more modern form of helping would be to institutionalize this within the state. But the state does not take on responsibility for helping those in need. We pay taxes every month, I have not been on sick leave since starting work and I have not been to a doctor, so I pay it for the system, and it turns out that it is not enough and the system does not work.” Corpo-Egoist, male, 30 y.o.

“A moral company is the one that earns the most money, offers the biggest salaries and delivers high quality products. There are other organizations and foundations that solve social and environmental problems.” Corpo-Egoist, male, 25 y.o.

## Discussion

There are several conclusions to be drawn. Firstly, and the most importantly, the present study confirmed not only the role of demographic, but also psychographic variables in segmentation studies. Findings from our study are in line with literature showing that sole demography has limited explanatory power of CSR-related attitudes and reactions (Pérez et al., 2015a). There is only one strong demographical relation found, concerning gender. Consistently with previous results (i.e., Meyers-Levy, 1989), our study showed that women are generally more sensitive to CSR than men, as women dominate in most open to CSR segments (Sensible Optimists and Sensitive Intellectuals) and men are majority in less open ones (Passive Poseurs, Excluded and Frustrated, Corpo-Egoists). Results for all other demographical variables are less clear. As far as age is concerned, among two most open to CSR segments one (Sensible Optimists) is relatively old and one (Sensitive Intellectuals) is relatively young. The oldest of extracted segments (Family Pragmatics) is indifferent to CSR, although the least positive attitudes are held by the youngest group. This reveals that age has some contribution in explaining differences in attitudes toward CSR, however, only in interaction with other, more psychological variables. It is even more visible for other demographical variables: the most CSR-positive and the most CSR-negative segments: Sensible Optimists and Corpo-Egoists, are of similar education and income.

In terms of the role of psychographic variables, consistently with previous research exploring relations between personal values and reactions to CSR (i.e., González-Rodríguez et al., 2016), more open to CSR segments usually prize self-transcedence and maturity, while those less open are more concerned about social status. However, the present study is the first to our knowledge that includes personal values in market segmentation of consumer reactions to corporate responsibility.

It is worth underlying that our findings not only proved the importance of including the psychographic variables in segmentation studies that focus on reactions on CRS, but also showed that personal values give more consistent results, comparing to demographic variables, and seem to be more helpful than demographic variables in understanding differences in behaviors and attitudes of the extracted segments. These findings serve as an important argument in the discussion of the role of psychological variables in CSR segmentation and give premise to include psychographic variables in segmentation studies in the future. Apart from personal values, such studies could also include other variables reported as positively related to prosocial behavior, such as social perspective taking (Eisenberg et al., 2001; Imuta et al., 2016), empathy (Hoffman, 2008), or altruism (Batson and Powell, 2003).

Another important conclusion is that in our study, due to including psychographic variable in the segmentation analysis and applying mixed-method approach, we were able to capture a variety of attitudes toward CSR among Polish consumers. Previous segmentation studies done in the area of attitudes to CSR or responsible consumption usually gradate the segments from most to least open to CSR ones (Chan, 2000; Pelsmacker et al., 2005; Vassilikopoulou et al., 2005; Mostafa, 2009; Finisterra do Paço and Raposo, 2010). A little more complicated structure has been found by Roberts (1995), who compared and contrasted two types of consumer behavior: social responsibility and ecological consciousness. However, two differing responsibly behaving consumer segments: Greens and Socially Responsible, were followed by two less distinct ones: Middle Americans, presenting both types of behavior at a medium level, and Browns, low at both. In contrast, in our study, we found considerable qualitative differences among all segments. As far as most open to CSR segments are concerned, on one hand, we have Sensible Optimists, rational and thoughtful, concentrated on the greater good, who value strategic and considerate actions by firms. On the other hand, there are Sensitive Intellectuals, who help out of an emotional need, value most causes that are personally relevant to them and expect companies to engage mostly for altruistic reasons. There are also great differences on the other pole – among CSR-negative segments. On one hand, there are Excluded and Frustrated, who often believe that firms should help others, but will never respond to CSR as consumers, because of their lack of knowledge or interest in the idea. On the other hand, there are Corpo-Egoists, with a relatively good understanding of what CSR is, but skeptical and mostly believing that firms should concentrate on selling good products and making good profits.

Final remark is about mixed method approach applied in the research. With few exceptions (Webb and Mohr, 1998; Siltaoja, 2006; Öberseder et al., 2011), the vast majority of studies in the area of CSR and consumer responsibility is quantitative, using experimental or correlational studies. Lack of contact with real consumer, giving him the possibility to speak up and explain motives of his behavior may sometimes lead to ambiguous and difficult to explain results. For example, Finisterra do Paço and Raposo (2010) in their segmentation of Portuguese consumers in terms of pro-ecological behaviors and attitudes, describe the segment of “Undefined,” presenting often conflicting behavior and attitudes, somewhat similar to Passive Poseurs found in our study. Qualitative interviews enabled us to verify their declarations. As it is quite easy to declare something untrue in quantitative survey, for instance that one often volunteers, it is more difficult to do so in face-to-face open-question interview, when moderator asks about specific actions, memories and feelings.

Qualitative interviews contributed to our research also in other ways. They gave us deeper and better understanding of values system of each segment, of how it is understood and realized in everyday life, which was exceptionally helpful in understanding the attitudes and behavior of Corpo-Egoists. Finally, last but not least important, they provided wider context enabling us to better understand where different attitudes to CSR come from. It is due to qualitative study that we know that Sensible Optimists are considerate and strategic in their engagement, while Sensitive Intellectuals are spontaneous caregivers or that Excluded and Frustrated are actually excluded and show little knowledge or interest in many areas of modern social politics and problem solving, not just CSR. To conclude, adding qualitative understanding to quantitative pattern of results is an important strength of our study and gives a new perspective in explaining people’s CSR-related behaviors. Finally, there are some limitations to the present study that must be pointed out. First, the decision of what variables would be included in the model, however, based on theoretical assumptions, was arbitrary and beyond doubt there are other important psychological or CSR-related factors that could be included and would change the shape of the segmentation. Second, the study was conducted in Poland and it is reasonable to assume that the results cannot be generalized to a wider population. Possibly they might reflect the situation in other eastern European countries, however, one must remember that despite a similar history, current political and economic circumstances of these countries are often very different, thus such generalizations should be made with great caution.

## Data Availability Statement

Datasets are in a publicly accessible repository. The datasets generated for this study can be found in the Open Science Framework repository, https://osf.io/wap2k/?view_only=c31c54af7e934e849d6f6bf1384fda3d.

## Ethics Statement

The studies involving human participants were reviewed and approved by Ethics Committee of the Faculty of Psychology, University of Warsaw. Written informed consent for participation was not required for this study in accordance with the national legislation and the institutional requirements.

## Author Contributions

DM contributed to the conception and design of the study and organized the database. AF was involved in performing the qualitative part of the study. DM, AF, and KS contributed to the statistical analysis of quantitative data. AF and DM performed the analysis of qualitative data. AF and KS wrote the first draft of the manuscript. All authors contributed to manuscript revision, read and approved the submitted version. AF – 35%, DM – 35%, and KS – 30% is total involvement of each author in creating the manuscript.

## Conflict of Interest

The authors declare that the research was conducted in the absence of any commercial or financial relationships that could be construed as a potential conflict of interest.
