# Supplementary material for: Segmentation Based on Attitudes Toward Corporate Social Responsibility in Relation to Demographical Variables and Personal Values – Quantitative and Qualitative Study of Polish Consumers
Source: Front Psychol. 2020 Mar 17;11:450. doi: 10.3389/fpsyg.2020.00450 (PMC7090225; doi:10.3389/fpsyg.2020.00450)
Supplement: Supplementary file 1 [file Table_1.DOCX]

# **Appendix – Survey questions**

**Q1. How often have you performed each of the listed activities in the last year?**

| Rotate | | **0 – never** | **1 – once** | **2 – a few times** | **3 – many times** | **4 – I do not know that kind of action** |
| --- | --- | --- | --- | --- | --- | --- |
| **A** | Make a payment to the foundation's account | 0 | 1 | 2 | 3 | 4 |
| **B** | Send SMS for charity | 0 | 1 | 2 | 3 | 4 |
| **C** | Donate on the street | 0 | 1 | 2 | 3 | 4 |
| **D** | Buy a product because part of the profit would be donated for a social purpose | 0 | 1 | 2 | 3 | 4 |
| **E** | Volunteer in a foundation / organisation | 0 | 1 | 2 | 3 | 4 |
| **F** | Buy an organic product, marked with a certificate | 0 | 1 | 2 | 3 | 4 |
| **G** | Buy a product because it has not been tested on animals | 0 | 1 | 2 | 3 | 4 |
| **H** | Take action to save resources (energy, water) | 0 | 1 | 2 | 3 | 4 |
| **J** | ‘Like’ a social or charitable action on social media (i.e., on Facebook) | 0 | 1 | 2 | 3 | 4 |
| **K** | Take part in an action where the consumer clicks on a button on a website so that the advertisers donate to a charity | 0 | 1 | 2 | 3 | 4 |
| **L** | Choose an offer from a firm that engages prosocially | 0 | 1 | 2 | 3 | 4 |
| **M** | Segregate waste | 0 | 1 | 2 | 3 | 4 |
| **N** | Take part in a brand/ company charitable/ social event | 0 | 1 | 2 | 3 | 4 |
| **P** | Shop with own multi-use bag | 0 | 1 | 2 | 3 | 4 |
| **R** | Choose product in environmentally friendly packaging | 0 | 1 | 2 | 3 | 4 |
| **S** | Choose a product a firm with a good reputation | 0 | 1 | 2 | 3 | 4 |
| **U** | Talk with family, friends about ethics of different firms’ activity | 0 | 1 | 2 | 3 | 4 |
| **V** | Buying a product for mainly ethical reasons (i.e., fair trade) | 0 | 1 | 2 | 3 | 4 |
| **X** | Opting out of receiving paper forms of invoices due to concern for the environment | 0 | 1 | 2 | 3 | 4 |

Q2. Have you ever heard of terms below:

| Rotate | | **I have heard of it and I know what it means** | **I have heard of it, but I don't know what it means** | **I haven't heard about the concept** |
| --- | --- | --- | --- | --- |
| **A** | Corporate social responsibility (CSR) | 1 | 2 | 3 |
| **B** | Fair Trade | 1 | 2 | 3 |
| **C** | Cause-Related Marketing (CRM) | 1 | 2 | 3 |
| **D** | Sustainable development | 1 | 2 | 3 |

**Q3. Below there are listed statements, defining different objectives of the existence of companies. Please read them all carefully and choose one that is closest to your personal opinion.**

| The main goal of companies is to make money – I do not expect any other action from them | 1 |
| --- | --- |
| Companies are primarily there to make money, but should also take some action on the environment, communities, etc. | 2 |
| Companies should actively support various activities for the benefit of society and the environment, e.g., allocating their money and time for those purposes | 3 |
| Companies should not only support, but also initiate various activities for the benefit of society and the environment, and actively seek solutions to solve various problems | 4 |

**Q4. What is your reaction when you hear that a company is engaged in societal or environmental issues?**

| I have positive feelings | 1 |
| --- | --- |
| I don't care | 2 |
| I have negative feelings | 3 |

**Q5. In your opinion, why do companies engage prosocially and proenvironmentally? Below there is a list of statements. Please select to what extent you agree with each of them.**

| Rotate | | **1 – I definitely do not agree** | **2 – I rather do not agree** | **3 – I rather agree** | **4 – I definitely do not agree** |
| --- | --- | --- | --- | --- | --- |
| **A** | Firms do it in order to enhance reputation and public image | 1 | 2 | 3 | 4 |
| **B** | They want to gain more trust | 1 | 2 | 3 | 4 |
| **C** | They want to reduce business expenses | 1 | 2 | 3 | 4 |
| **D** | They want the firm’s value to increase (i.e., so that the company's shares are listed higher on the stock exchange) | 1 | 2 | 3 | 4 |
| **E** | They want to gain new clients – ones that care about social and environmental issues | 1 | 2 | 3 | 4 |
| **F** | It helps them to develop new products and/or services | 1 | 2 | 3 | 4 |
| **G** | It helps to increase client loyalty (they more eagerly buy the products of such a company) | 1 | 2 | 3 | 4 |
| **H** | It increases the loyalty of the employees | 1 | 2 | 3 | 4 |
| **I** | It helps to build market competitiveness | 1 | 2 | 3 | 4 |
| **J** | It makes the company earn more – it turns into business profits | 1 | 2 | 3 | 4 |
| **K** | They just want to engage, solve the problem | 1 | 2 | 3 | 4 |
| **L** | They do it mainly for money, to earn more | 1 | 2 | 3 | 4 |

Q6. In this part of questionnaire please ask yourself „What values and principles are important to me?”

| Rotate | | **1 – definitely unimportant** | **2 – rather unimportant** | **3 –neither unimportant, nor important** | **4 – rather important** | **5 – definitely important** |
| --- | --- | --- | --- | --- | --- | --- |
| A | A world at peace | 1 | 2 | 3 | 4 | 5 |
| B | Wisdom (a mature understanding of life) | 1 | 2 | 3 | 4 | 5 |
| C | Justice | 1 | 2 | 3 | 4 | 5 |
| D | Tolerance | 1 | 2 | 3 | 4 | 5 |
| E | Protecting the environment | 1 | 2 | 3 | 4 | 5 |
| F | Being helpful | 1 | 2 | 3 | 4 | 5 |
| G | Personal security | 1 | 2 | 3 | 4 | 5 |
| H | Security of family and friends | 1 | 2 | 3 | 4 | 5 |
| I | National security | 1 | 2 | 3 | 4 | 5 |
| J | Social order | 1 | 2 | 3 | 4 | 5 |
| K | Health | 1 | 2 | 3 | 4 | 5 |
| L | Social power | 1 | 2 | 3 | 4 | 5 |
| M | Being influential (having an impact on people and events) | 1 | 2 | 3 | 4 | 5 |
| N | Social recognition | 1 | 2 | 3 | 4 | 5 |
| O | Choosing own goals | 1 | 2 | 3 | 4 | 5 |
| P | Ambition | 1 | 2 | 3 | 4 | 5 |
| R | Wealth | 1 | 2 | 3 | 4 | 5 |
| S | Enjoying life | 1 | 2 | 3 | 4 | 5 |
| T | Satisfying own needs | 1 | 2 | 3 | 4 | 5 |
| U | Looking for experience, adventure | 1 | 2 | 3 | 4 | 5 |
| V | Curiosity about the world | 1 | 2 | 3 | 4 | 5 |
| W | Independent (in thought and action) | 1 | 2 | 3 | 4 | 5 |
| X | Creativity | 1 | 2 | 3 | 4 | 5 |
| Y | Freedom | 1 | 2 | 3 | 4 | 5 |
| Z | Self-respect | 1 | 2 | 3 | 4 | 5 |
| AA | Taking care of family | 1 | 2 | 3 | 4 | 5 |
| AB | True friendship | 1 | 2 | 3 | 4 | 5 |
| AC | Love | 1 | 2 | 3 | 4 | 5 |
| AD | A spiritual life | 1 | 2 | 3 | 4 | 5 |
| AE | Respect for elders | 1 | 2 | 3 | 4 | 5 |
| AF | Humility | 1 | 2 | 3 | 4 | 5 |
| AG | Dutifulness | 1 | 2 | 3 | 4 | 5 |
| AH | Sense of belonging | 1 | 2 | 3 | 4 | 5 |
| AI | Reciprocity | 1 | 2 | 3 | 4 | 5 |
| AJ | Respect for others | 1 | 2 | 3 | 4 | 5 |
| AK | Responsibility | 1 | 2 | 3 | 4 | 5 |
| AL | Honesty | 1 | 2 | 3 | 4 | 5 |
| AM | Preserving my public image | 1 | 2 | 3 | 4 | 5 |
